# Normal Coronary Artery Patient Presenting with Left Ventricular Aneurysm

**DOI:** 10.1155/2011/183050

**Published:** 2011-08-07

**Authors:** Hakan Altay, Cihan Altin, Ali Çoner, Haldun Muderrisoglu

**Affiliations:** ^1^Department of Cardiology, Baskent University School of Medicine, Adana Hospital, Adana, Turkey; ^2^Department of Cardiology, Baskent University School of Medicine, Ankara Hospital, Ankara, Turkey

## Abstract

Left ventricular aneurysm (LVA) is one of the most important complications of myocardial infarction LVA is strictly defined as a distinct area of abnormal left ventricular diastolic contour with systolic dyskinesia or paradoxical bulging. LVA usually results from myocardial infarction. Other rare aetiologies of LVA include hypertrophic cardiomyopathy, Chagas' disease, sarcoidosis, congenital LVA, and idiopathic However, LVA formation in patients with idiopathic dilated cardiomyopathy is rarely reported, and the incidence, clinical features, and pathogenesis of LVA formation in patients with idiopathic dilated cardiomyopathy is not well understood. Here, we present a 45 years old, idiopathic dilated cardiomyopathy patient with LVA and normal coronary arteries The pathogenesis of LVA formation in patients with idiopathic dilated cardiomyopathy is not clear. One acceptable hypothesis is that coronary artery emboli originate from mural thrombi, present in some patients with idiopathic dilated cardiomyopathy, which develop due to local wall infarction and fibrosis. The local myocardial perfusion differences could be seen in idiopathic dilated cardiomyopathy and predominantly found in the anteroposterior axis of the left ventricle. Local fibrosis occurs more frequently on the anterior wall or posterior wall, and less frequently on the lateral or septal wall. In our patient, LVA existed in the septal segments.We could not define the exact mechanism of the septal aneurysm in our patient but we decided to present this abnormal case, which is different from cases thus far reported in the literature.

## 1. Introduction

Left ventricular aneurysm (LVA) is one of the most important complications of myocardial infarction and is thought to develop in five to ten percent of all patients with acute myocardial infarction [1]. Left ventricular aneurysm is defined as expansion of the dyskinetic area of the left ventricle wall. These aneurysms usually arise from a patch of weakened tissue in a ventricular wall. This, in turn, may block the passageways leading out of the heart, leading to severely constricted blood flow to the body. Ventricular aneurysms can be fatal if they rupture. 

Left ventricular aneurysms are generally related to myocardial infarction or coronary artery malformations but can also be associated with other rare causes, such as hypertrophic cardiomyopathy, Chagas' disease, sarcoidosis, and congenital or other idiopathic conditions. However, LVA formation in patients with idiopathic dilated cardiomyopathy is rarely reported, and the incidence, clinical features, and pathogenesis of LVA formation in patients with idiopathic dilated cardiomyopathy are not well understood [2]. Here, we present a case of LVA detected in an idiopathic dilated cardiomyopathy patient with normal coronary arteries.

## 2. Case

**Figure 1:**
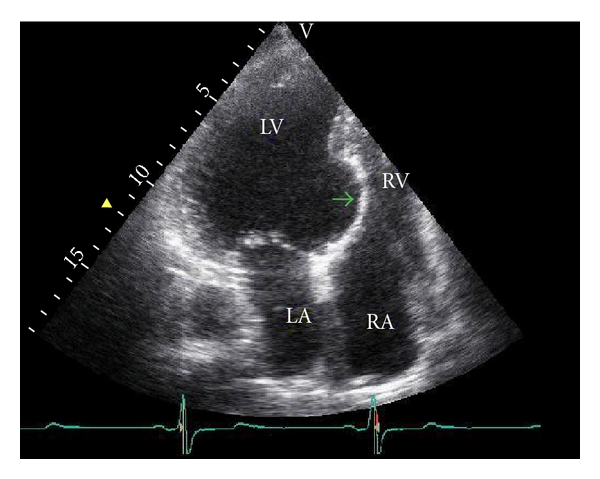
Transthoracic echocardiographic image of left ventricular septal aneurysm. LA: left atrium, RA: right atrium, LV: left ventricle, RV: right ventricle.

**Figure 2:**
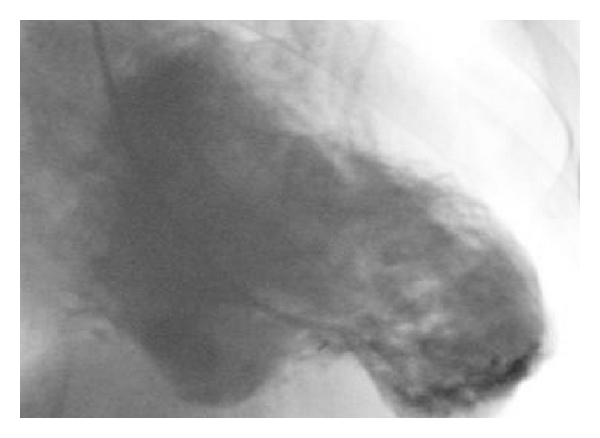
Ventriculographic image of left ventricular aneurysms.

**Figure 3:**
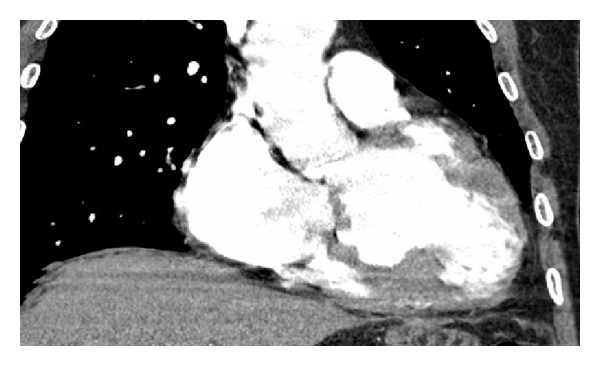
Cardiac computerized tomography image of left ventricular aneurysms.

A 45-year-old male was admitted to our cardiology clinic with exertional dyspnea and atypical chest pain complaints. He had no systemic diseases, no smoking history, and no family history of cardiovascular diseases. He had New York Heart Association (NYHA) Class II exertional dyspnea which had worsened over the course of two months. His blood pressure was 140/80 mmHg, and his pulse was 55 bpm. Upon cardiovascular examination, a third-degree systolic murmur was heard at apical and pulmonary foci. The rest of the systemic examination was normal. On his electrocardiography, 55 beats per minute sinus rhythm inferolateral nonspecific ST-segment depressions were detected. No Q wave was observed. On echocardiographic examination, his left ventricular ejection fraction was 31 percent, and the ventricle was severely and globally hypokinetic. The left heart cavities and ascending aorta were dilated. Aneurysmatic dilatation of the interventricular septum was observed (Figure 1). Coronary angiography revealed that the coronary arteries were normal. Ventriculography led to the detection of anterobasal and septal aneurysm, and the posterobasal segment was hypokinetic (Figure 2). On cardiac computerized tomography, three aneurysmatic dilatations were detected (the largest of aneurysmatic dilatation was measured 26 mm) (Figure 3). After consultation with the cardiovascular surgery department, we decided against surgery in this case. Instead, medical therapy for heart failure was planned.

## 3. Discussion

Left ventricular aneurysm is strictly defined as a distinct area of abnormal left ventricular diastolic contour with systolic dyskinesia or paradoxical bulging. LVA usually results from myocardial infarction. Other rare aetiologies of LVA include hypertrophic cardiomyopathy, Chagas' disease, sarcoidosis, congenital LVA, and idiopathic LVA [2]. However, LVA formation in patients with nonischaemic dilated cardiomyopathy (NIDCM) is rarely reported. In our patient, coronary angiography revealed no significant stenosis in the coronary arteries, which may exclude the possibility of ischaemic-dilated cardiomyopathy. However, if a complete or partial regression of coronary obstruction had occurred, then the coronary arteries could appear nearly normal at the time of an angiographic examination [3]. Thus, it may be possible that our patient had suffered myocardial infarction with normal coronary arteries. However, this is unlikely in light of the diffuse hypokinesis of the entire left ventricle wall seen in the patient. Furthermore, it cannot easily be accounted for by a local coronary event, and the location of the LVA did not appear to be related to the coronary distribution. Other possible etiologies of LVA, such as hypertrophic cardiomyopathy, Chagas' disease, sarcoidosis, congenital LVA, and idiopathic LVA could be ruled out based on the medical history, clinical examination, and laboratory test results. The pathogenesis of LVA formation in patients with NIDCM is not clear. One acceptable hypothesis is that coronary artery emboli originate from mural thrombi, present in some patients with NIDCM and/or LVA, which develop due to local wall infarction and fibrosis [1]. However, as our patient did not have any episodes of chest pain or ST-segment changes, nor did they have elevated cardiac enzymes suggestive of acute myocardial infarction, and since the location of the LVA was unrelated to the coronary artery distribution, the possibility of mural thrombus-derived emboli is unlikely. Although NIDCM diagnosis is defined on the basis of diffuse hypokinesis, regional variations in left ventricular contractility and myocardial perfusion are frequent in NIDCM. The regional asynergy seen in this patient may be related to the heterogeneity of local wall fibrosis and local wall stress [2]. 

Juillière et al. defined the local myocardial perfusion differences seen in NIDCM and found that they are predominant in the anteroposterior axis of the left ventricle. Local fibrosis occurs more frequently on the anterior wall or posterior wall, and less frequently on the lateral or septal wall [4]. Hayashida et al. showed that wall stress in patients with NIDCM was maximal in the basal segments and minimal in the apical segments [5]. In our patient, LVA existed in the septal segments, a condition which differs from otherwise similar NIDCM cases presenting with LVA [2, 6, 7]. We could not define the exact mechanism of the septal aneurysm in our patient, but we decided to present this abnormal case, which is different from cases thus far reported in the literature.
